# Driveline Infections Among Patients Supported With Left Ventricular Assist Devices: A Single Center Sixteen‐Year Longitudinal Profile

**DOI:** 10.1111/aor.70082

**Published:** 2025-12-21

**Authors:** Anh Nguyen, Alexis Shafii, Gabriel Loor, Andrew Civitello, O. Howard Frazier, Todd Rosengart, Kenneth Liao

**Affiliations:** ^1^ Division of Cardiothoracic Transplantation and Circulatory Support, Department of Surgery Baylor College of Medicine Houston Texas USA; ^2^ Division of Cardiology, Department of Medicine Baylor College of Medicine Houston Texas USA; ^3^ Cardiothoracic Surgery, Department of Surgery Baylor College of Medicine Houston Texas USA

**Keywords:** driveline infections, left ventricular assist device, mechanical circulatory support

## Abstract

**Background:**

Left ventricular assist device (LVAD) driveline infections significantly impact patient outcomes. This study aimed to identify their risk factors.

**Methods:**

We analyzed LVAD data from our institutional Intermacs database (January 1, 2008—December 31, 2023), combining primary implants and pump exchanges. Patient characteristics were summarized as frequencies for categorical variables and median (IQR) for continuous variables. The nature of repeated infection events was handled by the Andersen‐Gill method within a multivariable Cox proportional hazards model for cause‐specific hazard of driveline infections, accounting for death and heart transplant as competing risks.

**Results:**

Our cohort included 1026 LVAD implants. Median patient age was 57.7, and 79.6% were male. Cumulative driveline infection rates at 1, 2, 3, 4, and 5 years were 11.9%, 19.6%, 29.3%, 38.9%, and 40.1%, respectively, with a median time to infection of 13.8 months. Severe diabetes (HbA1c ≥ 8) increased driveline infection risk by 52% (HR = 1.52, *p* = 0.031). Pulsatile‐flow, fully magnetically levitated, and axial‐flow LVADs had a 245%, 70%, and 44% higher risk of driveline infections compared to partial magnetically levitated LVADs (HR = 3.45, *p* = 0.003; HR = 1.70, *p* = 0.066; HR = 1.44, *p* = 0.087, respectively). Paradoxically, patients 40 or above had over 58% lower risk of driveline infections than those under 40 (HR < 0.42, *p* < 0.001). In addition, coronary artery disease was associated with 56% lower risk of driveline infections (HR = 0.44, *p* = 0.001).

**Conclusions:**

Driveline infections remain prevalent in LVAD patients, especially those with severe diabetes. The lower driveline infection risk in older patients, those with coronary artery disease, and partially magnetically levitated LVADs warrants further investigation.

## Graphical abstract

Our study of 1026 LVAD implants revealed HeartWare had lower driveline infection rates than other LVAD types. In addition, severe diabetes was associated with higher risk, while older age and coronary artery disease were linked to a lower risk of driveline infections.

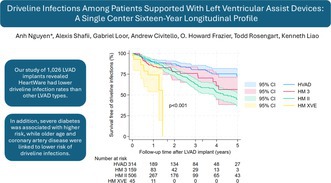

## 1 Introduction

Heart failure (HF) poses a significant global health issue, impacting over 64 million individuals worldwide and more than 6.7 million adults in the United States each year [1]. Despite progress in treatments, the outlook for patients with advanced HF remains bleak. As the population ages and the incidence of comorbidities like diabetes, hypertension, and hyperlipidemia rise, the prevalence of HF is anticipated to increase.

Heart transplantation continues to be a highly effective treatment for advanced heart failure, greatly enhancing quality of life and prolonging survival. However, the scarcity of donor hearts resulting in only around 3500 transplants per year in the U.S. means many patients remain in need. Left ventricular assist devices (LVADs) have become an essential option, providing short‐term support as a bridge to transplant or long‐term support [2].

Durable LVADs require a driveline to connect the external batteries to the internally implanted pump. This driveline, which passes through the skin, is susceptible to infection—a common complication that affects approximately 9.1% of LVAD recipients within the first 3 months post‐implant and 29.3% thereafter [3]. In this study, we assessed characteristics and risk factors of driveline infections in LVAD patients at Baylor St. Luke's Medical Center/Texas Heart Institute.

## 2 Methods

### 2.1 Data Source and Study Population

The Interagency Registry for Mechanically Assisted Circulatory Support (Intermacs) is a North American database that tracks clinical outcomes for patients who receive FDA‐approved mechanical circulatory support (MCS) devices for advanced heart failure. Intermacs collects longitudinal data throughout the patient's life with an MCS device. It is one of the four National Databases maintained by the Society of Thoracic Surgeons (STS). Currently, around 36 000 patients are enrolled from over 200 sites. Our institution regularly submits standardized data from our MCS patients to Intermacs and receives validated datasets on a quarterly basis.

We reviewed our institutional Intermacs database to identify all adult patients aged 19 and older who received durable LVADs, either as a primary device or through pump exchange, between January 1, 2008, and December 31, 2023. The implants were intended for either bridge‐to‐transplant or long‐term support. Patients who received temporary LVADs, right ventricular assist devices, or total artificial hearts were excluded from the study.

This study received approval from the Institutional Review Board for Human Subject Research at Baylor College of Medicine and Affiliated Hospitals, with a waiver of patient consent and HIPAA authorization (H‐51123, approved on November 29, 2022).

### 2.2 Statistical Analysis

Patient characteristics were reported as frequencies and proportions for categorical variables and as median and interquartile range (IQR) for continuous variables. Pearson chi‐square test was used to compare categorical variables; Wilcoxon rank sum test was used to compare continuous variables by driveline infection status. Univariable Cox proportional hazards regression was used to identify potential risk factors for driveline infections. Covariates were chosen based on medical literature. Variables were included in multivariable Cox proportional hazards regression model in a stepwise fashion based on their relative significance in univariable models (*p* < 0.1) or clinical judgment of our team. Correlated variables were not entered simultaneously. The final model included statistically significant covariates with *p* < 0.05 and/or clinically meaningful ones.

To account for repeated infection events, we employed the Andersen‐Gill method within a Cox proportional hazards model framework. The effect of covariates on the cause‐specific hazard of driveline infections was accounted for death and heart transplant as competing risks. The non‐independence of observations arising from patients with multiple devices by employing cluster‐robust standard errors using the variance–covariance estimator option in Stata. This approach adjusts the standard errors to allow for arbitrary correlation of residuals within each patient, which is essential given the presence of repeated measurements per individual. By clustering at the patient level, we obtain valid inference even in the presence of within‐patient correlation and heteroskedasticity, thereby improving the reliability of our statistical conclusions.

There is no missing data for variables included in the final model except for severe diabetes with missing rate less than 0.01% so no imputation was done. To assess the proportional‐hazards assumption, we used the assumption based on Schoenfeld residuals. The results indicated that the proportional‐hazards assumption was not violated for any of the covariates, as evidenced by non‐significant *p*‐values (*p* > 0.05) for each covariate. Model discrimination power was assessed with Harrell's censored C‐statistic with 1000 replication bootstrapping. Analyses were performed on Stata version 18.0 (Stata Corp LLC, College Station, TX, USA).

## 3 Results

### 3.1 Patient Characteristics

A total of 1026 LVADs were implanted during the study period, including both primary implants and pump exchanges. The median age was 57.7 years (IQR 47.3–65.1), and 817 patients (79.6%) were male. The majority (80.2%) had pre‐implant Intermacs profile ≤ 3 (high acuity). Pulsatile‐flow LVADs were used in 45 (4.4%) patients, axial‐flow LVADs in 508 (49.5%) patients, partially magnetically levitated centrifugal‐flow LVADs in 314 (30.6%) patients, and fully magnetically levitated centrifugal‐flow LVADs in 159 (15.5%) patients. All baseline characteristics by driveline infection status are presented in Table 1. The median follow‐up time was 13.8 months (IQR 4.2–32.5). The cumulative follow‐up time for the whole cohort was 1680 years.

**TABLE 1:** Patient characteristics with and without driveline infections.

	All patients (*n* = 1026)	Patients with driveline infections (*n* = 216)	Patients without driveline infection (*n* = 810)	*p*
Age, *N* (%)				
< 40	140 (13.6)	58 (26.9)	82 (10.1)	< 0.001
40–49	173 (16.9)	40 (18.5)	133 (16.4)	
50–59	281 (27.4)	60 (27.8)	221 (27.3)	
60–69	307 (29.9)	43 (19.9)	264 (32.6)	
> 70	125 (12.2)	15 (6.9)	110 (3.6)	
Female, *N* (%)	209 (20.4)	42 (19.4)	167 (20.6)	0.704
Black, *N* (%)	338 (32.9)	70 (32.4)	268 (33.1)	0.850
Intermacs profile < 4, *N* (%)	821 (80.0)	159 (73.6)	662 (81.7)	0.008
LVAD types, *N* (%)				
HM XVE	45 (4.4)	10 (4.6)	35 (4.3)	0.002
HM II	508 (49.5)	131 (60.7)	377 (46.5)	
HVAD	314 (30.6)	46 (21.3)	268 (33.1)	
HM 3	159 (15.5)	29 (13.4)	130 (16.1)	
Coronary artery disease, *N* (%)	300 (29.2)	33 (15.3)	267 (33.0)	< 0.001
Chronic kidney diseases, *N* (%)	422 (41.1)	72 (33.3)	350 (43.2)	0.009
Severe diabetes, *N* (%)	356 (34.7)	74 (34.3)	282 (34.9)	0.870
Body composition, *N* (%)				
Underweight	49 (4.8)	3 (1.4)	46 (5.7)	0.004
Normal weight	295 (28.7)	51 (23.6)	244 (30.1)	
Overweight	274 (26.7)	59 (27.3)	215 (26.5)	
Obesity	408 (39.8)	103 (47.7)	305 (37.7)	
Hospital dialysis, *N* (%)	81 (7.9)	7 (3.2)	74 (9.1)	0.04
Length of stay, median (IQR)	26 (18–41)	24 (17–37)	27 (18–42)	0.027
Concomitant surgery, *N* (%)	430 (41.9)	116 (53.7)	314 (38.8)	< 0.001

Abbreviations: HM 3, HeartMate 3, a fully magnetically levitated centrifugal‐flow LVAD; HM II, HeartMate II, an axial‐flow LVAD; HM XVE, HeartMate XVE, a pulsatile‐flow LVAD; HVAD, HeartWare, a partially magnetically levitated centrifugal‐flow LVAD; LVAD, Left ventricular assist device.

### 3.2 Driveline Infections After LVAD Implantation

Driveline infections were reported in 216 cases (21.1% or 9.4 per 100 person‐years), with 44.9% being reinfections. Driveline infection rates for pulsatile‐flow, axial‐flow, partially and fully magnetically levitated centrifugal‐flow LVADs were 22.2%, 25.8%, 14.7% and 18.2%, respectively. There were 119 cases (55.1%) with 1 driveline infection event, 60 (27.8%) with 2 events, 24 (11.1%) with 3 events, 8 (3.7%) with 4 events, and 5 (2.3%) with 5 events. The median time from LVAD implant to first driveline infection was 11.4 months (IQR 5.5–27.1). The median time from LVAD implant to any driveline infection was 17.0 months (IQR 6.7–33.3). The cumulative hazard of driveline infection at 1, 2, 3, 4, 5 years after LVAD implant was 11.9%, 19.6%, 29.3%, 38.9% and 40.1%. Table 1 presents characteristics of patients with and without driveline infections.

### 3.3 Risk Factors Associated With Driveline Infections

**FIGURE 1:**
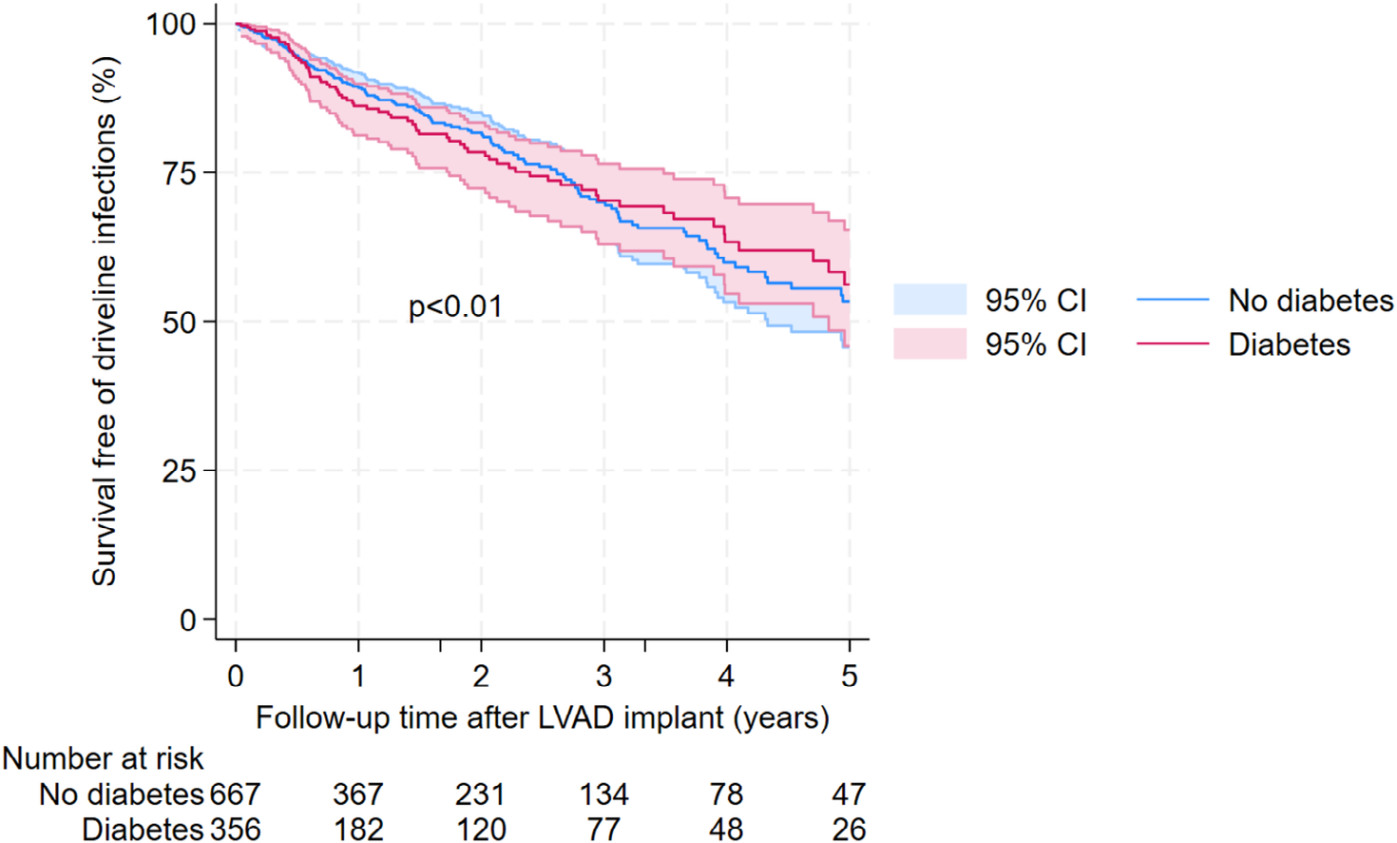
Survival free of driveline infection curves by status of severe diabetes. [Color figure can be viewed at wileyonlinelibrary.com]

**FIGURE 2:**
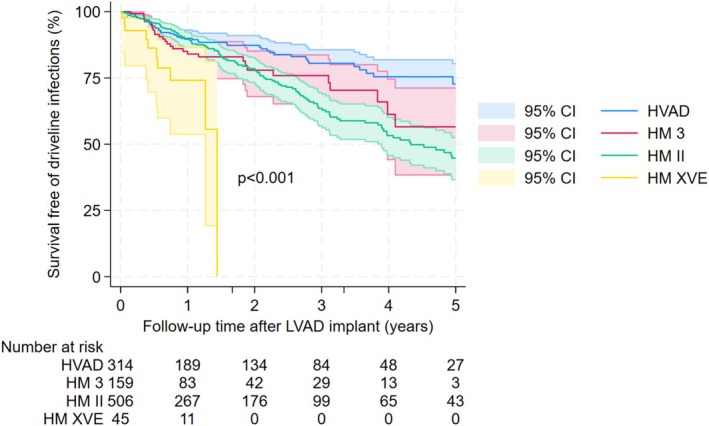
Survival free of driveline infection curves by LVAD types. [Color figure can be viewed at wileyonlinelibrary.com]

**FIGURE 3:**
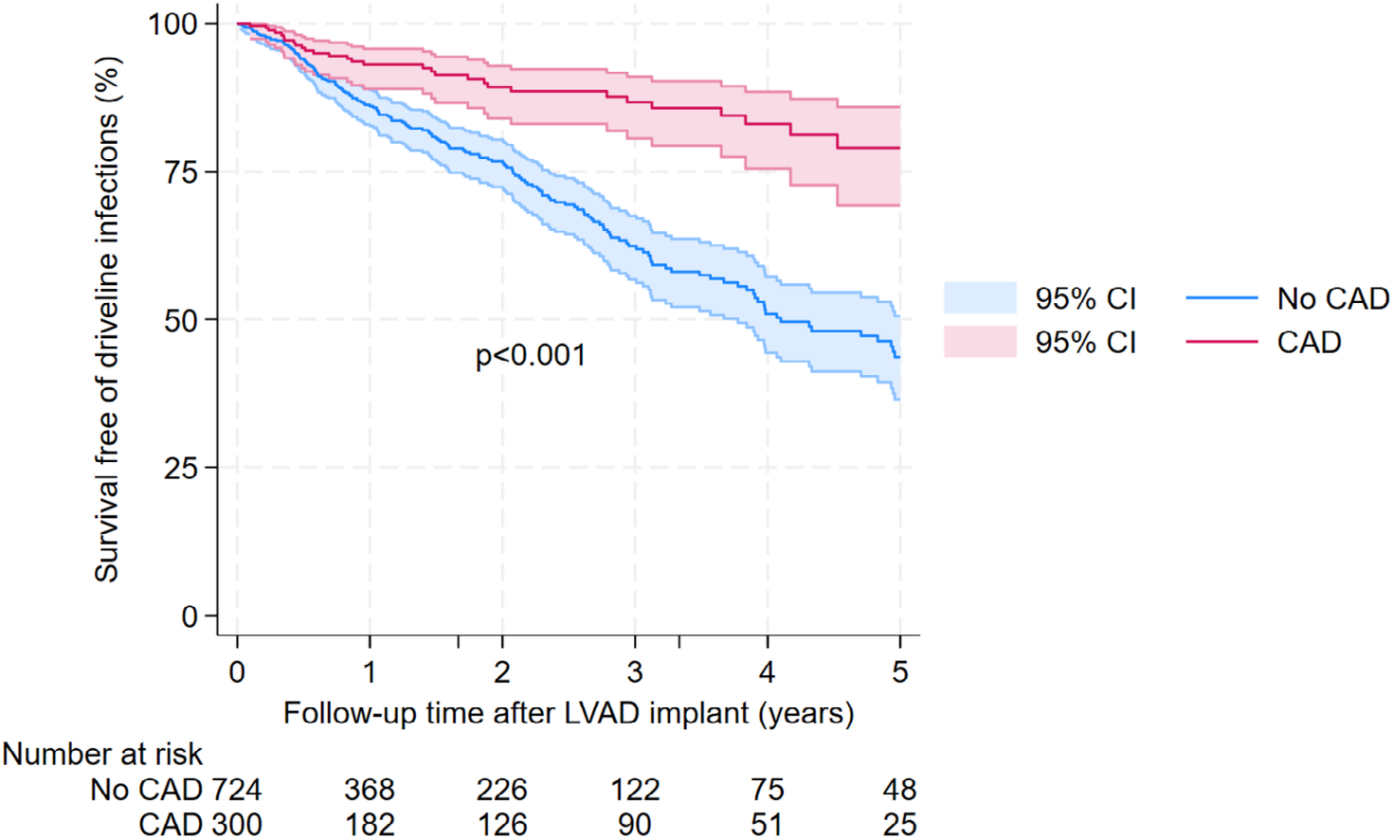
Survival free of driveline infection curves by status of coronary artery disease. [Color figure can be viewed at wileyonlinelibrary.com]

**FIGURE 4:**
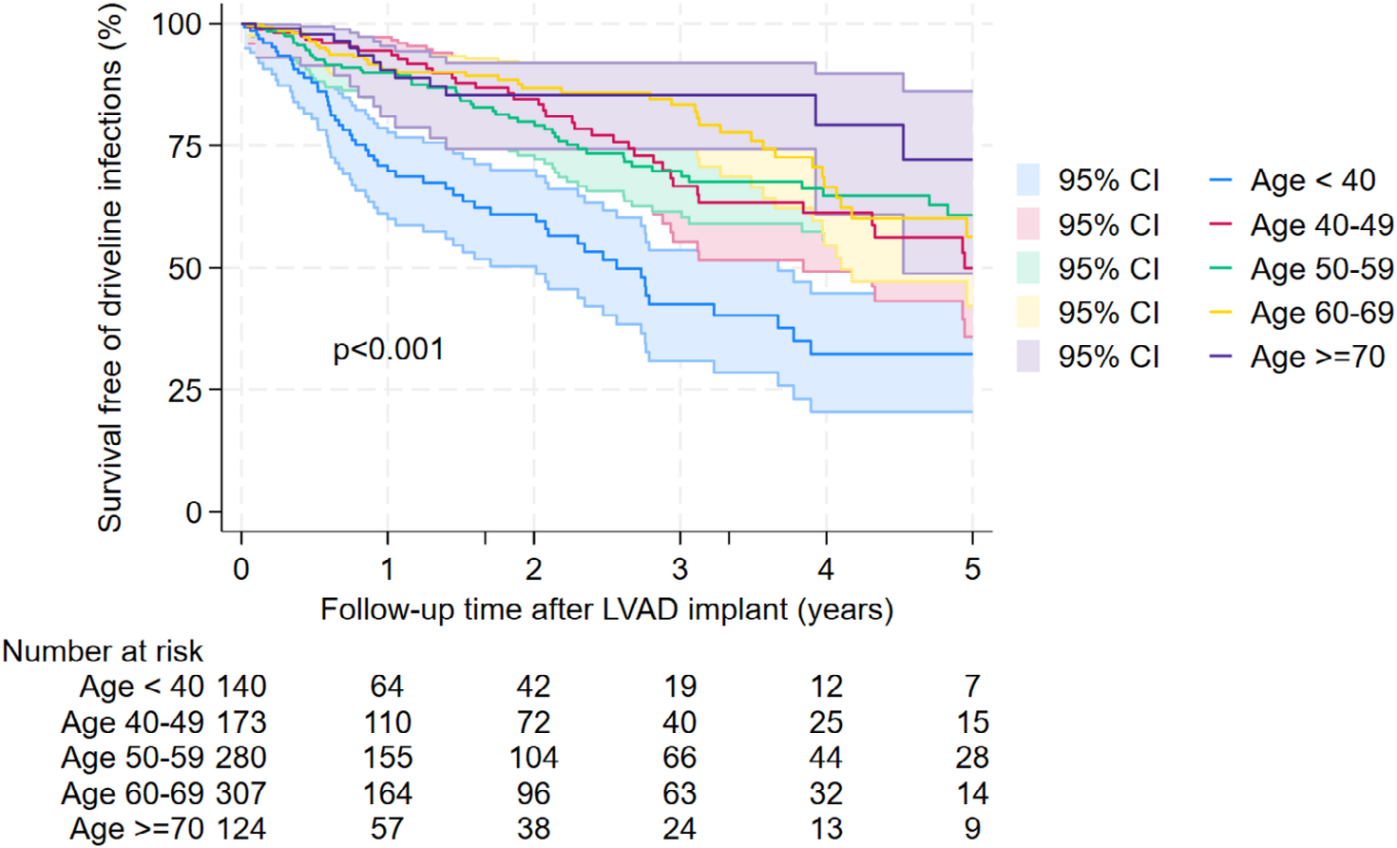
Survival free of driveline infection curves by age groups. [Color figure can be viewed at wileyonlinelibrary.com]

Table 2 presents univariable and multivariable Cox regression analysis results. Age, LVAD types, coronary artery disease, and severe diabetes were significantly associated with driveline infections after controlling for other potential confounders. Patients aged 40 or above had at least 58% lower risk of driveline infections as compared to those under 40 (adjusted HR < 0.42, *p* < 0.001). Pulsatile‐flow, fully magnetically levitated, and axial‐flow LVADs had a 245%, 70%, and 44% higher risk of driveline infections compared to partial magnetically levitated LVADs (adjusted HR = 3.45, *p* = 0.003; adjusted HR = 1.70, *p* = 0.066; adjusted HR = 1.44, *p* = 0.087, respectively). Patients with severe diabetes (HbA1c ≥ 8) had an increased driveline infection risk by 52% (adjusted HR = 1.52, *p* = 0.031). In addition, coronary artery disease was associated with 56% lower risk of driveline infections (adjusted HR = 0.44, *p* = 0.001). The curves for survival free of driveline infections by these four significant characteristics are presented in Figures 1, 2, 3, 4.

**TABLE 2:** Univariable and multivariable analysis of risk factors for driveline infections.

Characteristics	Univariable analysis			Multivariable analysis		
	Crude HR	95% CI	*p*	Adjusted HR	95% CI	*p*
Age						
< 40	Ref			Ref		
40–49	0.34	0.21–0.56	< 0.001	0.36	0.22–0.61	< 0.001
50–59	0.40	0.26–0.61	< 0.001	0.42	0.27–0.66	< 0.001
60–69	0.26	0.16–0.42	< 0.001	0.27	0.16–0.46	< 0.001
> 70	0.26	0.14–0.49	< 0.001	0.29	0.15–0.58	< 0.001
Female	0.91	0.62–1.35	0.654	0.85	0.55–1.32	0.462
Black	0.92	0.66–1.28	0.634	0.78	0.53–1.16	0.226
Intermacs profile < 4	0.75	0.52–1.08	0.123	0.73	0.49–1.08	0.115
LVAD types						
HM XVE	5.04	2.39–10.61	< 0.001	3.45	1.54–7.73	0.003
HM II	1.73	1.19–2.53	0.004	1.44	0.95–2.17	0.087
HVAD	Ref			Ref		
HM 3	1.64	0.99–2.70	0.054	1.70	0.97–2.98	0.066
CAD	0.34	0.23–0.53	< 0.001	0.44	0.27–0.71	0.001
CKD	0.73	0.53–1.01	0.060	1.03	0.70–1.52	0.881
Severe diabetes	1.00	0.73–1.40	0.967	1.52	1.04–2.21	0.031
Body composition						
Underweight	0.46	0.14–1.50	0.198	0.66	0.19–2.30	0.523
Normal weight	Ref			Ref		
Overweight	1.38	0.89–2.12	0.146	1.31	0.84–2.04	0.233
Obesity	1.33	0.89–1.99	0.163	1.03	0.67–1.58	0.896
Hospital dialysis	0.53	0.23–1.19	0.124	0.56	0.25–1.26	0.164
Length of stay						
Each 10‐day increase	0.99	0.93–1.04	0.665	1.01	0.96–1.07	0.650
Concomitant surgery	1.38	1.01–1.88	0.044	1.32	0.96–1.81	0.089

Abbreviations: CAD, coronary artery disease; CKD, chronic kidney disease; HM 3, HeartMate 3, a fully magnetically levitated centrifugal‐flow LVAD; HM II, HeartMate II, an axial‐flow LVAD; HM XVE, HeartMate XVE, a pulsatile‐flow LVAD; HVAD, HeartWare, a partially magnetically levitated centrifugal‐flow LVAD; LVAD, Left ventricular assist device.

Sex, race, Intermacs profile, chronic kidney disease, obesity, hospital dialysis, implant‐to‐discharge length of stay, and concomitant surgery were not statistically significant independent risk factors for driveline infections. But they were still kept in the final model due to their relevance according to literature and our team's clinical judgment. Chest closure was an important factor but not included due to its 88% missing data. Cardio‐pulmonary bypass was not included due to the small sample size (13) and too few events (3) in the off‐pump group. The discrimination of the model for GI bleeding was quite good, with a Harrell's C statistic of 0.70 (95% CI 0.65–0.74, *p* < 0.001).

**FIGURE 5:**
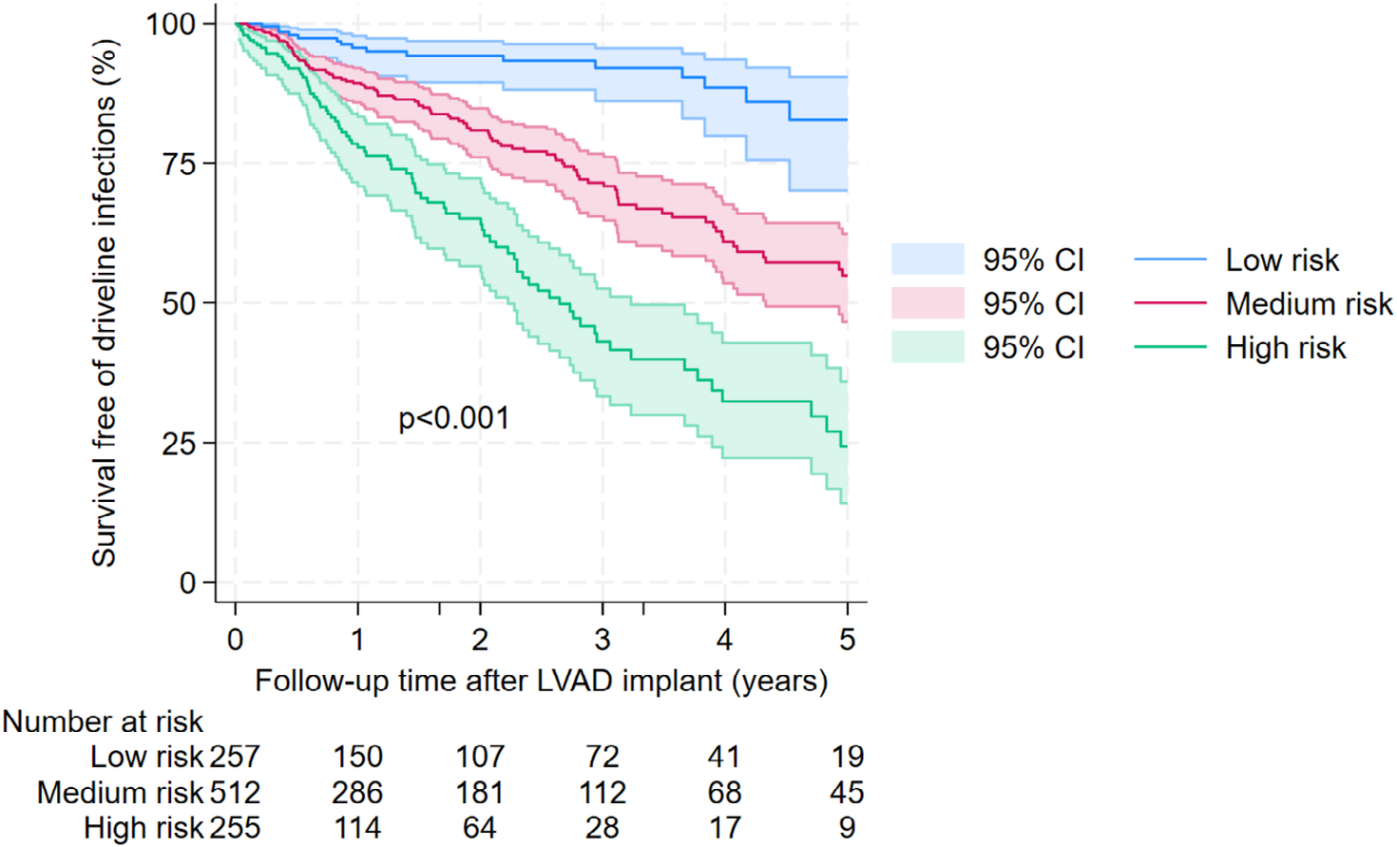
Survival free of driveline infection curves by risk categories. [Color figure can be viewed at wileyonlinelibrary.com]

We calculated a driveline infection risk score for each patient based on the final model and their corresponding characteristics. The patient cohort was classified into 3 categories: low (1st quartile), medium (2nd and 3rd quartiles), and high risk (4th quartile) groups per their corresponding risk scores. We presented the Kaplan Meier survival graph for driveline infections by these 3 risk categories in Figure 5. The log‐rank test with *p* < 0.001 showed a statistically significant difference in driveline infections among these groups for the whole follow‐up duration. The baseline characteristics of patients by risk categories are presented in Table S1.

## 4 Discussion

Our study described characteristics of driveline infections in a large LVAD cohort of 1026 patients at our institution. The driveline infection rates of 21.1% or 9.4 per 100 person‐years in our LVAD patients were lower than the registry statistics in ISHLT 2013–2015 IMACS report (14 per 100 person‐years) [3] and STS 2023 Annual Intermacs report (15–18 per 100 person‐years depending on reporting periods) [4]. According to a literature review, driveline infections reported by 34 studies could range from 0% to 92% depending on follow‐up durations and no meta‐analysis was done to pool an estimate [5] Our median time from LVAD implant to first driveline infections was 11.4 months (IQR 5.5–27.1), which was longer than 2.9 months (IQR 1.8–7.5) reported in a 3‐center study [6] and 6.5 months (IQR 3.1–12.4) reported in the 19‐center ASSIST‐ICD study [7].

Plausibly, we found severe diabetes to be a significant risk factor associated with increased driveline infections. It was consistent with findings in a study of 332 patients from the ADVANCE BTT and Continued Access Protocol trials [8]. However, three other single‐center studies did not find the significant association between diabetes and driveline infections, probably due to their small sample sizes [9, 10, 11].

We identified younger age as a significant risk factor of driveline infections after LVAD implantation similar to some other studies [12, 13]. We hypothesized that a higher activity for younger patients might have led to increased trauma to the driveline exit site and subsequently driveline infections. This might be the reason we could assume for the interesting observation that our LVAD patients with coronary artery diseases also had a significantly lower rate of driveline infections as compared to those without the condition.

Our study confirmed the higher rate of driveline infections among pulsatile‐flow LVADs as compared to continuous‐flow ones. It could be due to the fact that pulsatile‐flow LVADs have larger drivelines and other bulkier mechanical components that might make the exit sites more prone to trauma and infections. The HeartWare HVAD system is unique compared with other LVAD systems in that it has a thin (4.8 mm), flexible driveline and is implanted directly into the pericardial space without the need for a pump pocket [8]. It could be the reason we observed the lowest rate of driveline infections in patients with this LVAD type. In addition, the manufacturer of HeartWare might have a specialized cover material (Carbothane or Pellethane) designed to protect the driveline connection and prevent bacterial colonization, which is different from the manufacturer of HeartMate II and HeartMate 3. Interestingly, the rate of driveline infections was lower in axial‐flow (HeartMate II) as compared to fully magnetically levitated (HeartMate 3) LVADs. A possible reason could be that HeartMate 3 has a larger modular driveline (6.6 mm) than HeartMate II (6.0 mm) and is heavier and more difficult to stabilize with an anchor [14] Larger studies are needed to confirm our findings.

We could only utilize variables available in Intermacs database while other unmeasured or unavailable factors might affect the risk of driveline infections. They might include but are not limited to cardiopulmonary bypass, surgical approach, chest closure, sterile surgical technique, antibiotic prophylaxis, and post‐operative dressing care. Protocols and practice might change over time but with limitations, we assumed that these factors were not significantly different among LVAD patients with or without driveline infections at our institution at least for the relatively same time period in the 16 year duration of the study data. A prospective cohort study at Mayo Clinic showed that there was no significant difference in driveline infection rates between the groups with and without antibiotic prophylaxis [15]. Moreover, most driveline infections happened months after discharge when driveline care would be the role of patients and their families so practically future studies on driveline home care training would be beneficial. Priority should be given to those high risk of driveline infections as indicated by reliable predicting models such as ours in this study.

The ultimate solution to prevent driveline infections is getting rid of the drivelines themselves. Skin piercing by drivelines puts the patients at risk of infections. There are multiple technological advances in providing wireless power to LVADs, but there has not been a commercial product yet [16, 17, 18, 19]. In the meantime, preventing driveline infections, especially for those at higher risk, is still needed.

## 5 Conclusions

In this large cohort of LVAD patients at our institution in the past 16 years, driveline infections remain prevalent, especially in those with severe diabetes. The lower driveline infection risk in older patients, those with coronary artery disease, and partially magnetically levitated LVADs warrants further investigation to identify strategies to reduce the traumatic contact between the driveline and the skin that might lead to infections.

## Author Contributions

All authors contributed to the study conception and design. Material preparation, data collection, and analysis were performed by Anh Nguyen, Alexis Shafii, Gabriel Loor, Andrew Civitello, Kenneth Liao. The first draft of the manuscript was written by Anh Nguyen, and all authors commented on previous versions of the manuscript. All authors read and approved the final manuscript.

## Ethics Statement

The study (IRB number: H‐51123) is approved by Baylor College of Medicine institutional review board with the waiver of informed consents on November 29, 2022.

## Conflicts of Interest

O.H. Frazier, MD reports a relationship with BiVACOR that includes board membership and travel reimbursement. The other authors declare no conflicts of interest.

## Supporting information


**Table S1:** Patient characteristics by risk categories.

## References

[1] B. Bozkurt , T. Ahmad , K. Alexander , et al., “HF STATS 2024: Heart Failure Epidemiology and Outcomes Statistics an Updated 2024 Report From the Heart Failure Society of America,” Journal of Cardiac Failure 37, no. 3 (2024): 382–388, https://pubmed.ncbi.nlm.nih.gov/39322534/. 10.1016/j.cardfail.2024.07.001 39322534

[2] A. S. Varshney , E. M. DeFilippis , J. A. Cowger , I. Netuka , S. P. Pinney , and M. M. Givertz , “Trends and Outcomes of Left Ventricular Assist Device Therapy: JACC Focus Seminar,” Journal of the American College of Cardiology 79 (2022): 1092–1107. 35300822 10.1016/j.jacc.2022.01.017

[3] M. M. Hannan , R. Xie , J. Cowger , et al., “Epidemiology of Infection in Mechanical Circulatory Support: A Global Analysis From the ISHLT Mechanically Assisted Circulatory Support Registry,” Journal of Heart and Lung Transplantation 38, no. 4 (2019): 364–373. 10.1016/j.healun.2019.01.007 30733158

[4] U. P. Jorde , O. Saeed , D. Koehl , et al., “THE SOCIETY OF THORACIC SURGEONS INTERMACS ANNUAL REPORT THE Society OF Thoracic Surgeons Intermacs 2023 Annual Report: Focus on Magnetically Levitated Devices the 14th Annual Report From the Society of Thoracic Surgeons (STS) Interagency Registry for Mechanically Assisted,” Annals of Thoracic Surgery 117 (2024): 33–44, 10.1016/j.athoracsur.2023. 37944655

[5] N. V. Pavlovic , T. Randell , T. Madeira , S. Hsu , R. Zinoviev , and M. Abshire , “Risk of Left Ventricular Assist Device Driveline Infection: A Systematic Literature Review,” Heart & Lung 48, no. 2 (2019): 90–104, https://www.heartandlung.org/action/showFullText?pii=S0147956318303406. 30573195 10.1016/j.hrtlng.2018.11.002

[6] S. Siméon , E. Flécher , M. Revest , et al., “Left Ventricular Assist Device‐Related Infections: A Multicentric Study,” Clinical Microbiology and Infection 23, no. 10 (2017): 748–751. 28323195 10.1016/j.cmi.2017.03.008

[7] P. Tattevin , E. Flécher , V. Auffret , et al., “Risk Factors and Prognostic Impact of Left Ventricular Assist Device–Associated Infections,” American Heart Journal 214 (2019): 69–76. 31174053 10.1016/j.ahj.2019.04.021

[8] R. John , K. D. Aaronson , W. E. Pae , et al., “Drive‐Line Infections and Sepsis in Patients Receiving the HVAD System as a Left Ventricular Assist Device,” Journal of Heart and Lung Transplantation 33, no. 10 (2014): 1066–1073, https://pubmed.ncbi.nlm.nih.gov/25087103/. 10.1016/j.healun.2014.05.010 25087103

[9] C. O. Usoh , S. Sherazi , B. Szepietowska , et al., “Influence of Diabetes Mellitus on Outcomes in Patients After Left Ventricular Assist Device Implantation,” Annals of Thoracic Surgery 106, no. 2 (2018): 555–560. 29577927 10.1016/j.athoracsur.2018.02.045 PMC6057825

[10] A. R. Vest , S. M. Mistak , R. Hachamovitch , M. M. Mountis , N. Moazami , and J. B. Young , “Outcomes for Patients With Diabetes After Continuous‐Flow Left Ventricular Assist Device Implantation,” Journal of Cardiac Failure 22, no. 10 (2016): 789–796. 26924520 10.1016/j.cardfail.2016.02.010

[11] A. Rahal , Y. Ruch , N. Meyer , et al., “Left Ventricular Assist Device‐Associated Infections: Incidence and Risk Factors,” Journal of Thoracic Disease 12, no. 5 (2020): 2654–2662. 32642173 10.21037/jtd.2020.03.26 PMC7330372

[12] D. J. Goldstein , D. Naftel , W. Holman , et al., “Continuous‐Flow Devices and Percutaneous Site Infections: Clinical Outcomes,” Journal of Heart and Lung Transplantation 31, no. 11 (2012): 1151–1157, https://pubmed.ncbi.nlm.nih.gov/22766022/. 10.1016/j.healun.2012.05.004 22766022

[13] J. Bejko , F. Toto , D. Gregori , G. Gerosa , and T. Bottio , “Left Ventricle Assist Devices and Driveline's Infection Incidence: A Single‐Centre Experience,” Journal of Artificial Organs 21, no. 1 (2018): 52–60, https://pubmed.ncbi.nlm.nih.gov/28988400/. 28988400 10.1007/s10047-017-0997-y

[14] M. Kranzl , M. Stoiber , A. K. Schaefer , et al., “Driveline Features as Risk Factor for Infection in Left Ventricular Assist Devices: Meta‐Analysis and Experimental Tests,” Frontiers in Cardiovascular Medicine 8 (2021): 784208, www.frontiersin.org. 34977190 10.3389/fcvm.2021.784208 PMC8716483

[15] J. M. Stulak , S. Maltais , J. Cowger , et al., “Prevention of Percutaneous Driveline Infection After Left Ventricular Assist Device Implantation: Prophylactic Antibiotics Are Not Necessary,” ASAIO Journal 59, no. 6 (2013): 570–574, https://journals.lww.com/asaiojournal/fulltext/2013/11000/prevention_of_percutaneous_driveline_infection.5.aspx. 24172262 10.1097/MAT.0b013e3182a9e2a5

[16] H. Horie , T. Isoyama , and K. Ishiyama , “Design of a Hybrid Left Ventricular Assist Device With a New Wireless Charging System,” Artificial Organs 48, no. 3 (2024): 309–314, https://pubmed.ncbi.nlm.nih.gov/37877220/. 37877220 10.1111/aor.14666

[17] M. L. Karim , A. M. Bosnjak , J. McLaughlin , P. Crawford , D. McEneaney , and O. J. Escalona , “Transcutaneous Pulsed RF Energy Transfer Mitigates Tissue Heating in High Power Demand Implanted Device Applications: In Vivo and In Silico Models Results,” Sensors 22, no. 20 (2022): 36298125, https://pubmed.ncbi.nlm.nih.gov/36298125/. 10.3390/s22207775 PMC9611940 36298125

[18] H. Horie , T. Isoyama , and K. Ishiyama , “Design of an Innovative Wireless Left Ventricular Assist Device Driven by Either Extracorporeal Magnets or an Intracorporeal Battery Pack,” ASAIO Journal 69, no. 2 (2023): E73–9, https://pubmed.ncbi.nlm.nih.gov/36716071/. 36716071 10.1097/MAT.0000000000001874

[19] G. Monreal , S. C. Koenig , A. Sangwan , et al., “Feasibility Testing of the Bionet Sonar Ultrasound Transcutaneous Energy Transmission (UTET) System for Wireless Power and Communication of a LVAD,” Cardiovascular Engineering and Technology 15, no. 6 (2024): 39230796, https://pubmed.ncbi.nlm.nih.gov/39230796/. 10.1007/s13239-024-00748-9 PMC12318372 39230796

