# Supplementary material for: Driveline Infections Among Patients Supported With Left Ventricular Assist Devices: A Single Center Sixteen‐Year Longitudinal Profile
Source: Artif Organs. 2025 Dec 21;50(4):572–9. doi: 10.1111/aor.70082 (PMC13125384; doi:10.1111/aor.70082)
Supplement: Supplementary file 1 — Table S1: Patient characteristics by risk categories. [file AOR-50-572-s001.docx]

**TABLE S1. Patient characteristics by risk categories**

|  | **Low-risk (n=257)** | **Medium- risk**  **(n=513)** | **High-risk**  **(n=256)** | **p-value** |
| --- | --- | --- | --- | --- |
| **Age, N (%)** |  |  |  | <0.001 |
| <40 | 0 (0) | 27 (5.3) | 113 (44.1) |  |
| 40-49 | 37 (14.4) | 99 (19.3) | 37 (14.5) |  |
| 50-59 | 48 (18.7) | 153 (29.8) | 80 (31.3) |  |
| 60-69 | 125 (48.6) | 166 (32.4) | 16 (6.3) |  |
| >70 | 47 (18.3) | 68 (13.3) | 10 (3.9) |  |
| **Female, N (%)** | 43 (16.7) | 118 (23.0) | 48 (18.8) | 0.095 |
| **Black, N (%)** | 92 (35.8) | 174 (33.9) | 72 (28.1) | 0.145 |
| **Intermacs profile <4, N (%)** | 229 (89.1) | 401 (78.2) | 191 (74.6) | <0.001 |
| **LVAD types, N (%)** |  |  |  | <0.001 |
| HM XVE | 2 (0.8) | 6 (1.2) | 37 (14.5) |  |
| HM II | 69 (26.9) | 298 (58.1) | 141 (55.1) |  |
| HVAD | 116 (64.6) | 121 (23.6) | 27 (10.6) |  |
| HM 3 | 20 (7.8) | 88 (17.2) | 51 (19.9) |  |
| **Coronary Artery Disease, N (%)** | 201 (78.2) | 97 (18.9) | 2 (0.8) | <0.001 |
| **Chronic Kidney Diseases, N (%)** | 156 (60.7) | 192 (37.4) | 74 (28.9) | <0.001 |
| **Severe Diabetes, N (%)** | 89 (34.6) | 168 (32.8) | 99 (38.7) | 0.274 |
| **Body composition, N (%)** | |  |  | <0.001 |
| Underweight | 40 (15.6) | 5 (1.0) | 4 (1.6) |  |
| Normal weight | 88 (34.2) | 162 (31.6) | 45 (17.6) |  |
| Overweight | 51 (19.8) | 137 (26.7) | 86 (33.6) |  |
| Obesity | 78 (30.6) | 209 (40.7) | 121 (47.3) |  |
| **Hospital Dialysis, N (%)** | 49 (19.1) | 23 (4.5) | 9 (3.5) | <0.001 |
| **Length of stay, median (IQR)** | 28 (18-41) | 27 (19-41) | 24 (17-38) | 0.158 |
| **Concomitant surgery, N(%)** | 74 (28.8) | 221 (43.1) | 135 (52.73) | <0.001 |

LVAD : Left ventricular assist device

HM XVE : HeartMate XVE, a pulsatile-flow LVAD

HM II : HeartMate II, an axial-flow LVAD

HVAD : HeartWare, a partially magnetically levitated centrifugal-flow LVAD

HM 3 : HeartMate 3, a fully magnetically levitated centrifugal-flow LVAD
